# Letter to the Editor: Best Practices on Public and Patient Involvement in Interprofessional Healthcare Education

**DOI:** 10.1111/hex.70053

**Published:** 2024-10-04

**Authors:** Ricardo J. O. Ferreira, Matilde Leal, Elsa Frazão Mateus, Lucija Gosak, Matthijs H. Bosveld, Cathy C. Kline, the PULPIT Consortium, Cristina Baixinho, Adriana Henriques, Andreia Silva Costa, Paulo Costa, Catarina Lima, Pedro Morgado, Nadine Santos, Dante Mulder, Sjim Romme, Koen Goffings, Bruno Van Koeckhoven, Barbara Kegl, Mateja Lorber, Danielle Derijcke, Mitchell Silva, Angela Towle, Khadidja Abdallah, Isabelle Huys, Charlotte Verbeke

**Affiliations:** ^1^ Nursing Research, Innovation and Development Centre of Lisbon (CIDNUR), Nursing School of Lisbon (ESEL) Lisbon Portugal; ^2^ Faculdade de Medicina, Instituto de Saúde Ambiental (ISAMB) Universidade de Lisboa Lisbon Portugal; ^3^ Rheumatology Department Unidade Local de Saúde de Coimbra Coimbra Portugal; ^4^ Núcleo de Investigação em Enfermagem (NIE), Unidade Local de Saúde de Coimbra, a Clinical Unit of the Health Sciences Research Unit: Nursing (UICISA: E), Nursing School of Coimbra (ESEnfC) Coimbra Portugal; ^5^ Laboratório Associado TERRA, Faculdade de Medicina Universidade de Lisboa Lisboa Portugal; ^6^ European Patients' Academy on Therapeutic Innovation (EUPATI) Lisbon Portugal; ^7^ Liga Portuguesa Contra as Doenças Reumáticas (LPCDR) Lisboa Portugal; ^8^ Comprehensive Health Research Centre (CHRC) Lisbon Portugal; ^9^ Faculty of Health Sciences University of Maribor Maribor Slovenia; ^10^ Department of Family Medicine, Care and Public Health Research Institute (CAPHRI) Maastricht University Maastricht The Netherlands; ^11^ School of Health Professions Education (SHE) Maastricht University Maastricht The Netherlands; ^12^ Patient & Community Partnership for Education, Office of UBC Health University of British Columbia Vancouver Canada; ^13^ Escola de Medicina da Universidade do Minho Braga Portugal; ^14^ Stichting Mens achter de Patiënt Eijsden The Netherlands; ^15^ Hogeschool PXL Hasselt Belgium; ^16^ EUPATI Belgium Belgium; ^17^ Katholieke Universiteit Leuven Leuven Belgium; ^18^ Nursing Research, Innovation and Development Centre of Lisbon (CIDNUR), Nursing School of Lisbon (ESEL) Lisbon Portugal; ^19^ Patient & Community Partnership for Education, Office of UBC Health University of British Columbia Vancouver Canada; ^20^ Faculty of Health Sciences University of Maribor Maribor Slovenia

Dear Editor,

We read with interest the article ‘Public Participation in Healthcare Student Education: An Umbrella Review’ by Nowell et al. [1]. Their work highlights the benefits of involving patients in healthcare education, such as enhancing empathy, patient‐centred decision‐making and safety [1].

The PULPIT Consortium, funded by ERASMUS+, promotes public and patient involvement (PPI) in the interprofessional education (IPE) of healthcare students [2]. Our project addresses students' limited early patient interaction and poor understanding of patient‐centred care and healthcare roles. We aim to implement an educational module that will be freely accessible through a dedicated online platform, as well as recommendations for PPI in the IPE of undergraduate healthcare students, following the ‘Vancouver Statement’ [3]. This project builds upon two main partner initiatives: the ‘Health Mentors Programme’, coordinated by the Patient and Community Partnership for Education (PCPE; https://health.ubc.ca/pcpe), and the ‘Patient as a Person’ project [4], developed by the Maastricht University and the Patient as a Person Foundation (https://mensachterdepatient.nl/), which have been instrumental in advancing patient involvement in healthcare education.

Nowell et al.'s article [1] is a valuable resource for raising awareness of the benefits and complexities of PPI in healthcare education. Despite the wealth of research on the benefits, *authentic* patient involvement in IPE remains a blind spot. We challenge interprofessional educators to involve ‘Experts by Experience’ (EBEs) in all aspects of IPE (curriculum design, delivery, research and evaluation) so that students can learn how to collaborate with the public and patients as equal and valued members of the healthcare team. It is long overdue and is the core purpose of the PULPIT Consortium.

## Author Contributions

**Ricardo J. O. Ferreira:** conceptualization, supervision, writing–original draft, funding acquisition. **Matilde Leal:** writing–original draft, project administration. **Elsa Frazão Mateus:** writing–review and editing. **Lucija Gosak:** writing–review and editing. **Matthijs H. Bosveld:** conceptualization, writing–review and editing. **Cathy C. Kline:** conceptualization, writing–review and editing.

## The PULPIT Consortium

The members of the PULPIT Consortium include the following: Cristina Baixinho, Adriana Henriques, Andreia Silva Costa, Paulo Costa (Escola Superior de Enfermagem de Lisboa, Lisbon, Portugal); Catarina Lima, Pedro Morgado, Nadine Santos (Escola de Medicina da Universidade do Minho, Braga, Portugal); Dante Mulder, Sjim Romme (Stichting Mens achter de Patiënt, Eijsden, The Netherlands); Koen Goffings, Bruno Van Koeckhoven (Hogeschool PXL, Hasselt, Belgium); Barbara Kegl, Mateja Lorber (Univerza v Mariboru, Maribor, Slovenia); Danielle Derijcke, Mitchell Silva (EUPATI Belgium, Belgium); Angela Towle (University of British Columbia, Vancouver, Canada); Khadidja Abdallah, Isabelle Huys, Charlotte Verbeke (Katholieke Universiteit Leuven, Leuven, Belgium).

## Conflicts of Interest

The authors declare no conflicts of interest.

## Data Availability

The authors have nothing to report.
